# Pituitary Gland and Neurological Involvement in a Case of Hemophagocytic Syndrome Revealing an Intravascular Large B-Cell Lymphoma

**DOI:** 10.1155/2019/9625075

**Published:** 2019-04-28

**Authors:** Sylvain Raoul Simeni Njonnou, Bruno Couturier, Yannick Gombeir, Sylvain Verbanck, France Devuyst, Georges El Hachem, Ivan Theate, Anne-Laure Trepant, Virginie De Wilde, Frédéric-Alain Vandergheynst

**Affiliations:** ^1^Department of Internal Medicine and Specialties, Faculty of Medicine and Biomedical Sciences, University of Yaounde I, Yaoundé, Cameroon; ^2^Department of Internal Medicine, Hôpital Erasme, Université Libre de Bruxelles, Brussels, Belgium; ^3^Department of Endocrinology, Hôpital Erasme, Université Libre de Bruxelles, Brussels, Belgium; ^4^Institute of Pathology and Genetics, Gosselies, Charleroi, Belgium; ^5^Department of Pathology, Hôpital Erasme, Université Libre de Bruxelles, Brussels, Belgium; ^6^Department of Hematology, Hôpital Erasme, Université Libre de Bruxelles, Brussels, Belgium

## Abstract

Intravascular large B-cell lymphoma is a rare entity characterized by the proliferation of neoplastic lymphocytes in the lumen of small blood vessels and high mortality. Diagnosis of intravascular lymphoma is often delayed or established postmortem. Here, we report the case of a 48-year-old woman presenting hemophagocytic syndrome, with pituitary gland and neurological involvement. Diagnosis of intravascular large B-cell lymphoma was made on perisplenic vessels, while liver and bone marrow biopsy was noncontributive. This case demonstrates the importance of thorough histopathologic investigations in the setting of high suspicion.

## 1. Introduction

Intravascular large B-cell lymphoma (IVLBCL) is defined as a rare subtype of diffuse extranodal large B-cell lymphoma [1]. It is characterized by proliferation of neoplastic cells in the lumen of small blood vessels in several organs including the skin, central nervous system, and endocrine system [2–4]. The reason of this peculiar localization is not known. Given the fact that IVLBCL does not produce a mass or lymphadenopathy, the diagnosis is often nonevoked or is particularly difficult to confirm. Another pitfall lies in the variety of clinical presentation, which includes fever of unknown origin.

Hemophagocytic syndrome (HPS) is a severe disease, caused by phagocytosis of hematopoietic cells by activated macrophages not controlled by the usual immune system regulation. It is classified into primary (familial) or secondary forms (associated with infections or malignancies or systemic diseases or immunodeficiencies). Identification and treatment of the underlying etiology of secondary HPS is critical for treatment and prognosis [5].

Here, we present a case of IVLBCL in an immunosuppressed patient (treated by methotrexate and etanercept for seronegative rheumatoid arthritis) who was referred to the emergency department for fever and bicytopenia.

## 2. Case Presentation

**Figure 1:**
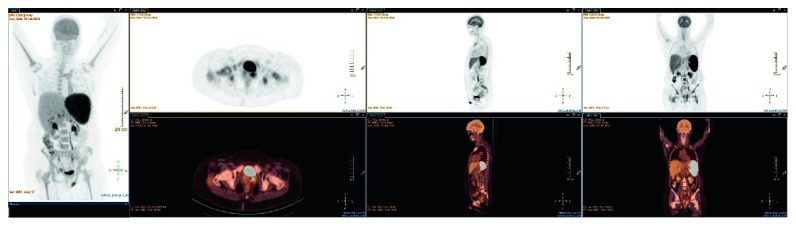
PET-CT hypermetabolic splenomegaly, perihepatic hypermetabolic lymph nodes, and cervical hypermetabolism.

A 48-year-old woman was referred to the emergency department with a one-week history of fever and cytopenia (Hb 8.7 g/dL, normal range 11.8–15.5; platelet count 77 × 10^9^/L, normal range 155–346). Clinical examination found a huge splenomegaly, confirmed by abdominal CT scan but no arthritis. 18F-fluorodeoxyglucose positron emission tomography/computed tomography (^18^FDG PET-CT) showed an enlarged hypermetabolic spleen and perisplenic and hilar hepatic hypermetabolic lymph nodes with hypermetabolism around the cervix (Figure 1). Biological investigations revealed mild anemia (Hb 8.4 g/dL), severe thrombopenia (platelet count 40 × 10^9^/L), elevated CRP (200 mg/L, normal range <10), acute liver cytolysis (GPT 57 U/L, normal range <34), hyperferritinemia (ferritin 4724 *μ*g/L, normal range 30–350), increased LDH (4482 U/L, normal range <214), increased soluble CD25 or soluble IL-2 receptor (>30000 pg/mL, normal range 632–4883), and biological intravascular disseminated coagulation (PT<50%, normal range >70% and fibrinogen 77 mg/dL, normal range 160–400). H-score, according to those results, was 99.6% [6, 7]. All this pattern was suggestive of HPS [8, 9]. Bone marrow aspiration and biopsy initially did not confirm and were not contributive for the etiology of HPS. All the infectious serologies including HTLV 1 and 2, HHV 6 and 8, parvovirus B19, *Toxoplasma*, and *Borrelia* were negative. EBV PCR and CMV PCR were negative. Evolution was marked by apparition of jaundice, worsening of liver function (total and conjugated bilirubin, alkaline phosphatase, gamma glutamyl transferase, and glutamic oxaloacetic transaminase were 4–6 times above normal range), and unexplained lactic acidemia (lactacte 8.9 mmol/L, normal range <2 mmol/l) without any clinical manifestation. Liver biopsy was performed, finding cholestasis without any inflammation probably due to toxic etiology. Etoposide (2 courses: first 300 mg and then 200 mg eight days later) and dexamethasone were given with good evolution. The patient was discharged on reduced doses of prednisone, with prednisone 8 mg/day planned to be gradually tapered.

**Figure 2:**
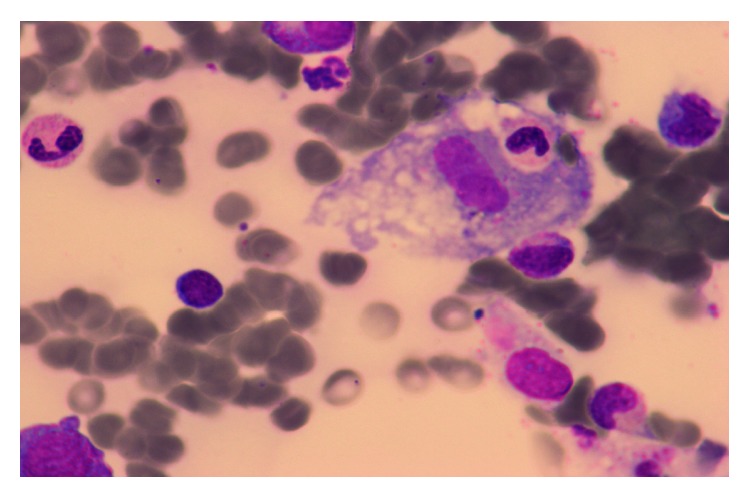
Hemophagocytosis at bone marrow aspiration.

**Figure 3:**
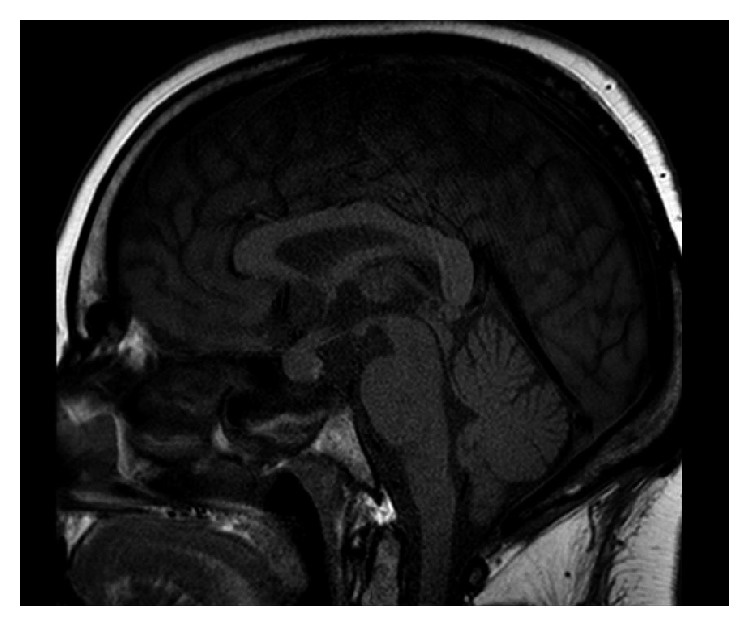
Brain MRI showing enlargement of the pituitary gland and pituitary stalk without evidence of adenoma.

**Figure 4:**
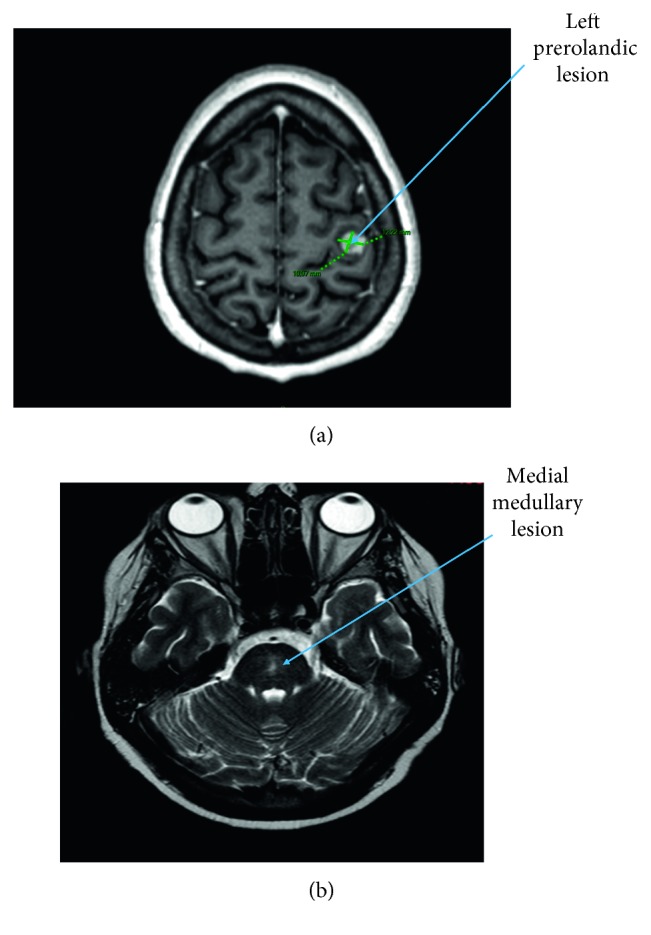
Brain MRI showing left prerolandic lesion and medial medullary hypersignal. (a) Prerolandic lesion with homogeneous corticosubcortical ring enhancement. (b) Medial medullary hypersignal in T2 and flair.

She was readmitted 3 weeks after discharge and 1 week after discontinuation of prednisone, for nausea and vomiting in the setting of inflammatory syndrome (CRP 117 mg/L). Clinical examination was unremarkable. New biological investigations were suggestive of HPS (sCD25 at 15000 pg/mL and ferritin at 1740 *μ*g/L) with evidence of hemophagocytosis at bone marrow aspirates, with acute renal failure and hypopituitarism (Figure 2), clearly concerning gonadotropic axis and thyrotropic axis. Gonadotropic hormone levels were as follows: estradiol <25 ng/L, progesterone <0.2 mcg/L (corresponding to menopause values), LH 2.5 IU/L (normal range: 7.7–58.5), and FSH 7 IU/L (normal range: 25.8–134.8). FSH and LH levels were inappropriate regarding progesterone and estradiol levels, although the patient was not taking oral contraceptives. Regarding thyrotropic axis, TSH was 0.1 mU/L (normal range: 0.3–4.2); free T4, 0.5 ng/dL (normal range 0.9–1.7); and free T3, 1.8 pmol/L (normal range 3.1–6.8). Growth hormone was 0.68 mcg/L (normal range 0.06–6.88), and IGF-1 dosage was performed. Corticotropic axis seemed to be normal: cortisol was 350 nmol/L in the morning (normal range 166–507) and ACTH was 11.4 ng/L (normal range 7.2–63.3). Finally, there was no diabetes insipidus. Brain MRI presented an enlarged pituitary gland and pituitary stalk with nonspecific lesions (Figure 3). A new ^18^FDG PET-CT showed a reduction of size and hypermetabolism of the spleen but no pituitary hypermetabolism. Levothyroxine and hydrocortisone substitution were started, the latter considered given the concern of an increased steroid need due to pyrexia and the diagnostic splenectomy rapidly planned. A rapid onset of paraparesis with urinary and fecal incontinence led us to perform a new brain MRI. It showed a hypersignaling (T2/flair) medial medullary lesion associated with corticosubcortical prerolandic homogeneous lesion (Figure 4). Analysis of CSF showed an inflammatory liquid with increased protein (0.80 g/L normal < 0.40) but normal cell count (2 cells/mL).

Given the result of histological examination, the patient has been treated by two courses of R-CHOEP with a three-week interval (rituximab 375 mg/m^2^; cyclophosphamide 750 mg/m^2^; doxorubicin 50 mg/m^2^; vincristine 1.4 mg/m^2^; etoposide 100 mg/m^2^ on day 1, 2, and 3; and methylprednisolone 80 mg/day), together with weekly intrathecal infusion of methotrexate 15 mg, cytarabine 40 mg, and methylprednisolone 40 mg. Under this treatment, a marked decrease in the size of the pituitary gland and pituitary stalk was noted. Nevertheless, two months after beginning this treatment, the patient died from several infections in the context of persistent medullary toxicity. No autopsy was performed.

## 3. Pathological Findings

**Figure 5:**
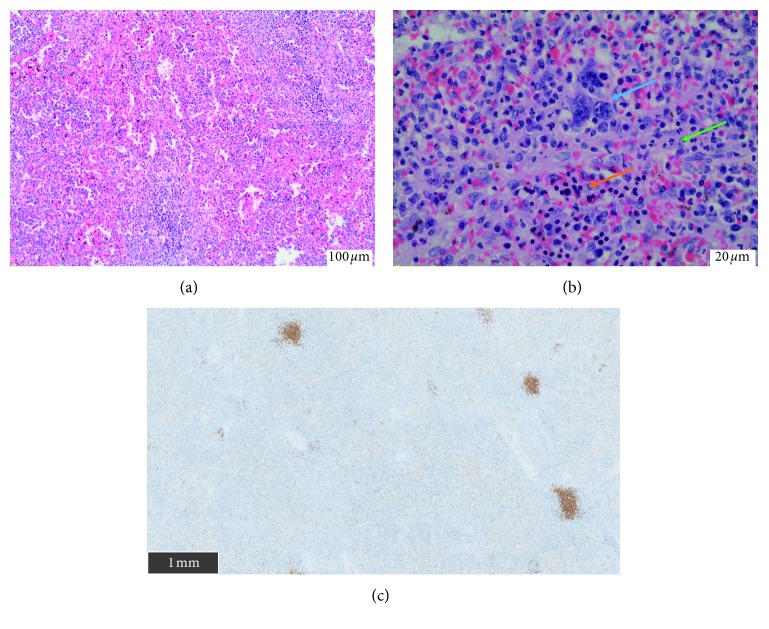
Histopathology of a splenectomy piece. (a) Extramedullary hematopoiesis (H&E, 10x). (b) Extramedullary hematopoiesis (H&E, 40x) with megakaryocytes (blue arrow), erythroblasts (orange arrow), and neutrophils (green arrow). (c) CD20 immunostaining showing no spleen infiltration (scale on image).

**Figure 6:**
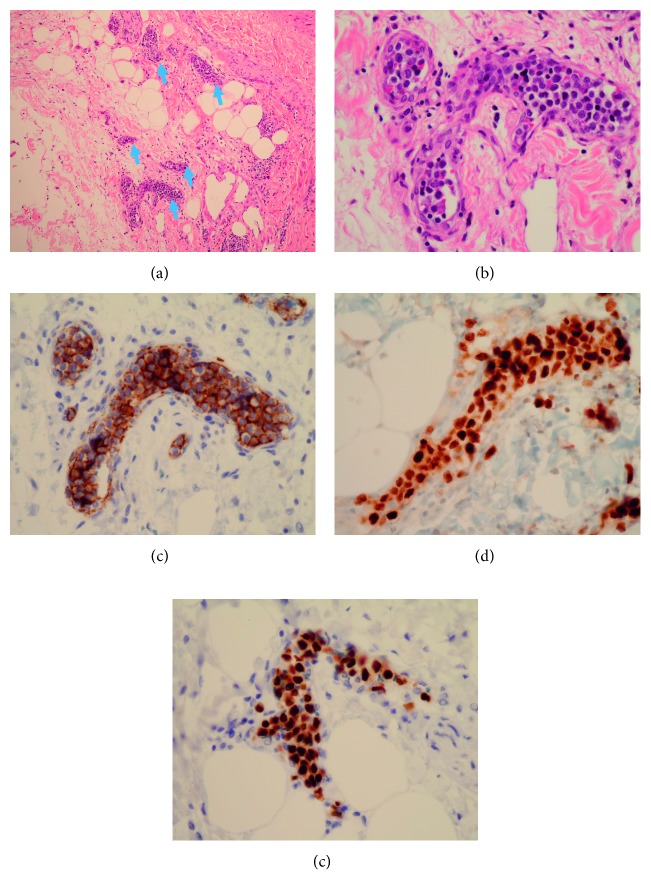
Histopathology of perisplenic tissues. (a) Tumoral intravascular infiltration (arrows) (H&E, 10x). (b) Large hyperchromatic cell inside small vessels around splenic lymph nodes (H&E, 40x). (c) Strong positive CD20 B-cell surface expression. (d) Positive expression of MUM1. (e) Positive expression of PAX5.

Histological examination revealed the following:

  At the spleen level: extramedullary hematopoiesis (with megakaryocytes, erythroblasts, and neutrophils). There was no intravascular infiltration by IVLBCL in the splenic tissue (Figure 5).
  At the periganglionic tissue level around the spleen: large atypical cells and hyperchromatic nucleolus, present inside the lumen of small vessels. These morphologic features were consistent with IVLBCL features. On immunostaining, tumor cells were histologically positive for CD20, PAX5, and MUM1 (Figure 6). EBV research was negative by “EBER (Epstein–Barr virus-encoded small RNA) in situ hybridization.”

## 4. Discussion

We describe a case of IVLBCL with neurologic symptoms and hypopituitarism occurring in a patient treated for seronegative rheumatoid arthritis. At the beginning, we considered the hypothesis of adult-onset Still's disease (AOSD) to explain both the HPS and the seronegative rheumatoid arthritis. But the lack of fever and elevated inflammatory parameters at the onset of arthritis a few years ago were not consistent with this hypothesis.

IVLBCL is a rare disease. It has been mainly described in the setting of case reports or short case series [4, 10, 11]. Its association with HPS has been reported several times [2, 12, 13]. Neurological involvement, mainly the central nervous system (CNS), represents 52% of the presentations. CNS is the main affected site. Cognitive impairment (61%), motor impairment (22%), and seizures (13.4%) are the most frequent CNS signs [14]. CNS localization of IVLBCL on the one hand and HPS on the other hand are confounding [3, 5]. It is difficult to affirm that the neurologic impairment in this patient is directly related to IVLBCL rather than HPS without any histological examination. However, given the rather good control of HPS for 3 months (on the basis of blood count), the neurological impairment is more likely related to the lymphoma than HPS. The treatment given for HPS—consisting in two courses of etoposide and steroids (first high-dose dexamethasone and then methylprednisolone)—had probably an impact on the clinical course of IVLBCL.

Pituitary gland involvement due to IVLBCL is exceptionally diagnosed on the basis of clinical and biological findings and rather evidenced postmortem [4, 15–18]. It could be isolated or associated in some cases with involvement of other endocrine glands (adrenal gland and ovaries) [4, 10, 18, 19]. The reason of the preferential tropism of clonal lymphocytes for the endocrine gland is not known. A transsphenoidal biopsy of the pituitary gland is not routinely performed because of the obvious risk of inducing or increasing panhypopituitarism. However, it is not established that pituitary involvement has an impact on the prognosis of patient [14]. Findings of pituitary imaging in case of hypopituitarism associated with IVLBCL vary from suprasellar mass, sellar mass, partially empty sella, or normal [20]. Despite the absence of pathological findings, the marked reduction in the size of the pituitary gland and pituitary stalk with treatment would be in favor of IVLBCL-related disease. Cases of complete resolution of pituitary involvement under treatment have been described [18–20].

However, pituitary gland involvement related to HPS, although exceptional, has been described in some case reports. The usual manifestation is diabetes insipidus, which is lacking in this case [21].

The most effective treatment of IVLBCL involving the CNS is a combination of R-CHOP and intrathecal methotrexate, which could even be administered in prophylaxis of CNS involvement [22]. Etoposide has been administered in relapse protocol in some patients, but in this case, the choice was driven by the association with HPS [23]. Overall survival of patients with CNS involvement of IVLBCL, treated (with classical treatment) or not, is very poor (<1 year), but complete remission has been reported in some cases [14, 18, 19]. Autologous hematopoietic stem cell transplantation is reported to be successful in almost all cases [24].

## 5. Conclusion

The particularity of our patient is the association of endocrine and neurologic involvement in the context of HPS. This case supports the recommendation of a thorough etiological workup in an adult HPS patient.
